# P2X4 Receptor *in Silico* and Electrophysiological Approaches Reveal Insights of Ivermectin and Zinc Allosteric Modulation

**DOI:** 10.3389/fphar.2017.00918

**Published:** 2017-12-15

**Authors:** Verónica Latapiat, Felipe E. Rodríguez, Francisca Godoy, Felipe A. Montenegro, Nelson P. Barrera, Juan P. Huidobro-Toro

**Affiliations:** ^1^Laboratorio de Farmacología de Nucleótidos, Departamento de Biología, Facultad de Química y Biología, Estación Central, Universidad de Santiago de Chile, Santiago, Chile; ^2^Departamento de Fisiología, Facultad de Ciencias Biológicas, Pontificia Universidad Católica de Chile, Santiago, Chile; ^3^Centro Desarrollo de Nanociencia y Nanotecnología, CEDENNA, Estación Central, Universidad de Santiago de Chile, Santiago, Chile

**Keywords:** P2X4R, positive allosteric modulation, ivermectin, Zn(II), molecular docking, molecular dynamics, independent allosteric modulator sites

## Abstract

Protein allosteric modulation is a pillar of metabolic regulatory mechanisms; this concept has been extended to include ion channel regulation. P2XRs are ligand-gated channels activated by extracellular ATP, sensitive to trace metals and other chemicals. By combining *in silico* calculations with electrophysiological recordings, we investigated the molecular basis of P2X4R modulation by Zn(II) and ivermectin, an antiparasite drug currently used in veterinary medicine. To this aim, docking studies, molecular dynamics simulations and non-bonded energy calculations for the P2X4R in the apo and holo states or in the presence of ivermectin and/or Zn(II) were accomplished. Based on the crystallized *Danio rerio* P2X4R, the rat P2X4R, P2X2R, and P2X7R structures were modeled, to determine ivermectin binding localization. Calculations revealed that its allosteric site is restricted to transmembrane domains of the P2X4R; the role of Y42 and W46 plus S341 and non-polar residues were revealed as essential, and are not present in the homologous P2X2R or P2X7R transmembrane domains. This finding was confirmed by preferential binding conformations and electrophysiological data, revealing P2X4R modulator specificity. Zn(II) acts in the P2X4R extracellular domain neighboring the SS3 bridge. Molecular dynamics in the different P2X4R states revealed allosterism-induced stability. Pore and lateral fenestration measurements of the P2X4R showed conformational changes in the presence of both modulators compatible with a larger opening of the extracellular vestibule. Electrophysiological studies demonstrated additive effects in the ATP-gated currents by joint applications of ivermectin plus Zn(II). The C132A P2X4R mutant was insensitive to Zn(II); but IVM caused a 4.9 ± 0.7-fold increase in the ATP-evoked currents. Likewise, the simultaneous application of both modulators elicited a 7.1 ± 1.7-fold increase in the ATP-gated current. Moreover, the C126A P2X4R mutant evoked similar ATP-gated currents comparable to those of wild-type P2X4R. Finally, a P2X4/2R chimera did not respond to IVM but Zn(II) elicited a 2.7 ± 0.6-fold increase in the ATP-gated current. The application of IVM plus Zn(II) evoked a 2.7 ± 0.9-fold increase in the ATP-gated currents. In summary, allosteric modulators caused additive ATP-gated currents; consistent with lateral fenestration enlargement. Energy calculations demonstrated a favorable transition of the holo receptor state following both allosteric modulators binding, as expected for allosteric interactions.

## Introduction

P2XRs are ATP-gated ionotropic channels; seven different clones are known which form functional channels as homo- and heterotrimers (Nicke et al., 1998; Barrera et al., 2005; Marquez-Klaka et al., 2007). Each subunit has two membrane domains and a large extracellular loop comprising 10 conserved cysteines to form five intersubunit disulphide bonds (Kawate et al., 2009). These receptor sets firmly established the extracellular role of nucleotides as novel signal molecules implicated in physiological and pathophysiological conditions including transmitter function in sympathetic co-transmission, the pain pathway, gliotransmission, epithelia and endothelial cell signaling, platelet aggregation, urinary reflex, smooth muscle contractility, bone physiology, among other roles (Burnstock, 2007; Köles et al., 2007; Coddou et al., 2011).

Upon ATP binding to its orthosteric site, conformational changes occur in the region linking the extracellular domain with the transmembrane (TM) helices, and the three lateral portals known as “lateral fenestrations”, which reach 8 Å in diameter in the holo state (Jiang et al., 2013). Analysis of the crystallized P2X4R structure (Hattori and Gouaux, 2012) revealed two possible cation pathways in the holo state via lateral fenestrations, or the central pore pathway lined by negatively charged residues. Recent experiments based on accessibility studies and on electrostatic energy calculations convincingly demonstrated that ions gain access to the P2X4R pore mainly through the three lateral fenestrations (Kawate et al., 2011; Samways et al., 2011, 2012; Roberts et al., 2012).

P2XRs, like other ionic ligand-gated channels, are modulated by several endogenous compounds, including trace metals such as Zn(II) or Cu(II) (Coddou et al., 2003b, 2005; Huidobro-Toro et al., 2008). Zn(II) is stored in synaptic vesicles. Upon electrical depolarization, a fraction of the stored metal may be released to the synaptic cleft (Kardos et al., 1989; Kay, 2006; Kay and Tóth, 2006) to modify neurotransmission (Peralta and Huidobro-Toro, 2016). Therefore, the final response due to the activation of the ATP-gated channels depends not only on the concentration of ATP, but also on the presence of allosteric modulators in the synapse. Since P2XR channels do not contain a linear metal binding motif, we hypothesized that the metal binding site forms after receptor protein folding, exposing the three-dimensional metal binding site. In the P2X4R, the specific role of C132 and H140 were identified related to the coordination of Zn(II) and Cu(II), respectively (Acuña-Castillo et al., 2000; Coddou et al., 2003a, 2007; Lorca et al., 2005; Huidobro-Toro et al., 2008), which are critical as structural elements to understand receptor topology and its molecular basis of allosteric modulation (Cully et al., 1994; Vassilatis et al., 1997; Adelsberger et al., 2000). Ivermectin (IVM), a semisynthetic macrocyclic lactone derived from *Streptomycetes avermectilis* is an antiparasite amply used in veterinary medicine (Omura and Crump, 2004; Geary, 2005). IVM has multiple ionic channel targets (including glutamate and nicotinic receptors), but in parasites it appears to paralyze nematodes by intensifying GABA-A-mediated peripheral nerve transmission. Several reports also indicate that IVM facilitates selectively P2X4R-mediated ATP-gated currents (Khakh et al., 1999; Priel and Silberberg, 2004). In the absence of IVM, this channel activates and deactivates rapidly, does not show transition from the open to dilated states, desensitizes completely at a moderate rate, and recovers only fractionally during washout. IVM treatment triggers a larger ATP-dependent current in a concentration and time-dependent manner, and slows receptor desensitization during sustained ATP applications and receptor deactivation. Rescuing the receptor from desensitization temporally coincides with pore dilation, and the dilated channel can be reactivated after ATP washout (Khakh et al., 1999; Priel and Silberberg, 2004; Jelínková et al., 2006; Zemkova et al., 2015). The spatial location of the IVM binding site has not yet been addressed in the context of the recent crystal structures of a zP2X4R in an apo, closed channel state (Kawate et al., 2009), and holo, open state, with ATP bound crystals (Hattori and Gouaux, 2012). The molecular mechanism of IVM action is related to an allosteric interaction that involves both TM helices at the protein-lipid interface during the opening of the P2X4R pore (Jelínková et al., 2006; Jelinkova et al., 2008; Silberberg et al., 2007; Zemkova et al., 2007). Mutations of nonpolar residues in the lower part of TM1 and TM2 helices apparently did not disturb IVM affinity, but rather the efficacy with which IVM potentiates the receptor. Additionally, the three-dimensional models of the IVM and TM1 fragment of P2X4R suggest that these residues might be accessible simultaneously by large IVM concentrations. In agreement with this information, none of the single mutants in this region fully eliminated IVM effects on current amplitude and the rate of deactivation (Silberberg et al., 2007; Jelinkova et al., 2008; Zemkova et al., 2014). Using molecular docking, W46, and W50 in TM1 plus D331, M336 in TM2 were identified as sites for IVM binding, while residues N338, S341, G342, L346, G347, A349, and I356 all belonging to the P2X4R TM2 which were supposed to play a role in IVM modulation (Silberberg et al., 2007; Jelinkova et al., 2008). Interestingly, these sites are located at the bottom of the P2X4R lateral fenestrations. However, the conformational changes induced by P2X4R positive allosteric modulators such as IVM or Zn(II) remain to be understood in detail.

We hypothesized that IVM and Zn(II) modulate independently the P2X4R by binding at separate sites that may cause additive or even synergistic effects when applied in a concomitant manner. To this aim, we combined *in silico* methods with electrophysiological protocols, to describe how and where these modulators elicit P2X4R positive allosterism. Based on P2X4R models, docking studies, and molecular dynamics (MD) simulations, pore and lateral fenestrations analysis and non-bonded energy were calculated. We assessed the mode of action of these modulators by studying the nature of the interaction in several P2X4R mutants and a P2X4/2R chimera. MD simulations of P2X4R bound to allosteric modulators are consistent with the opening of a larger upper extracellular vestibule and receptor lateral fenestrations, revealing the energetically favorable state transition induced by allosterism. The present report shows how receptor structural determinants provide pharmacodynamically relevant molecular insights to identify allosteric regulatory mechanisms.

## Materials and methods

### Molecular modeling of rat P2XRs

While rat P2X4R shares a 62% sequence identity with the open/closed state, *Danio rerio* P2X4R X-ray diffraction structure (PDB ID: 3I5D, Kawate et al., 2009; PDB ID: 4DW1, Hattori and Gouaux, 2012), P2X2R shares a 48% sequence identity with open state *D. rerio* P2X4R X-ray diffraction structure (PDB ID: 4DW1, Hattori and Gouaux, 2012), and P2X7R shares a 76% sequence identity with open state *Ailuropoda melanoleuca* P2X7R X-ray diffraction structure (PDB ID:5U2H, Karasawa and Kawate, 2016). Using these reference structures, we built homology models of P2X4R holo, P2X4R apo, P2X2R holo and P2X7R holo states, via Modeller v 9.10 software (Sali and Blundell, 1993). Final Modeler models were chosen based on model quality using the DOPE (Discrete Optimized Protein Energy) method (Shen and Sali, 2006), a statistical potential optimized for model assessment. Models were further assessed by RAMPAGE (Lovell et al., 2002) through Ramachandran plots for residue distribution in favored, allowed, and outlier regions. The energetic quality of three-dimensional models was verified by ProSA (Protein Structure Analysis) (Wiederstein and Sippl, 2007) to calculate an overall quality score of the predicted structures.

The rat P2X4R models were embedded into a phosphatidylcholine (POPC) lipid bilayer in a water box; hydrated systems were neutralized with 150 mM NaCl. The all-atom systems were subjected to MD simulations under periodic bordering conditions and isobaric-isothermal ensemble (NPT). The full systems were minimized using NAMD 2.9 software (Phillips et al., 2005) for 50,000 time-step minimization and subsequently equilibrated for 5 ns.

### Molecular docking of P2XRs with IVM

Homology minimized models of the rat P2X4 apo, P2X4R, P2X2R, and P2X7R holo states were used in docking simulations to identify IVM binding sites. The molecular docking procedure used Autodock software (Morris et al., 2009) employing the Lamarckian genetic algorithm (Morris et al., 1998). ATP and IVM structures were obtained from the Pubchem database (Kim et al., 2016). P2XR models were prepared using Autodock with grid size for ATP: 126 Å × 126 Å × 126 Å, and IVM: 120 Å × 120 Å × 80 Å). ATP and IVM binding sites were defined from known P2XR structures (PDB ID: 4DW1, Hattori and Gouaux, 2012; Popova et al., 2013). The rat P2XRs were kept rigid and ligands set flexible to rotate and explore the most probable binding poses. The search was done with 100 dockings with four repetitions per complex.

### MD simulations of allosteric modulators bound to P2X4Rs

Six systems were considered: P2X4R apo; P2X4R holo; P2X4R holo with IVM; P2X4R holo with Zn(II), and P2X4R holo with IVM plus Zn(II). MD simulations were performed with the CHARMM (Brooks et al., 2009) force field protocol, and each molecular complex was embedded into a 140 × 140 Å POPC lipid bilayer in a water box of the TIP3P water model (Jorgensen et al., 1983). The hydrated system was neutralized with 150 mM NaCl. The system was submitted to an MD simulation under periodic bordering conditions, NPT ensemble, and 300 K temperature. The full system was minimized by NAMD 2.9 software (Phillips et al., 2005) for 50,000 minimization steps, 5 ns thermodynamic equilibration, and 35 ns of production dynamics.

#### Pore radius and lateral fenestration measurements of P2XRs bound to allosteric modulators

After MD simulations of each system, the HOLE program (Smart et al., 1996) was used to determine P2X4R channel pore dimensions, considering the last 10 ns of trajectory simulation (see above). Lateral fenestration length was measured between Cα of D58 residues (Jiang et al., 2013) in adjacent receptor subunits. Similarly, the upper region of lateral fenestration was calculated using the distance between Cα of V323 and S62 residues from adjacent subunits.

#### Non-bonded energy calculations for P2X4R bound to allosteric modulators

Non-bonded energy (E_Non−bonded_ = E_van der Walls_ + E_Electrostatic_) (Levitt, 2001) was used to determine whether the reactions are energetically favorable (non-bonded energy of products lower than the energy of reactants) or not at constant pressure and temperature (Thauer et al., 1977). Non-bonded energy difference (ΔE_protein_), between the apo and holo state of P2X4R was calculated in the presence of allosteric modulators using the last 10 ns of the simulation trajectory (total simulation time 40 ns).

### Protocols characterizing P2XR expression

#### Oocyte microinjection and P2XR expression in *Xenopus laevis*

Stage 5–6 *Xenopus laevis* oocytes were manually defolliculated and incubated for 15 min with 1 mg/mL collagenase 1A. Separate sets of oocytes were injected intranuclearly with 3–5 ng cDNA for wild type (wt) rat P2X4R, P2X2R, or P2X7Rs, Cys-mutated P2X4R, and the P2X4/2R chimera receptor. Non-injected oocytes did not evoke currents upon exogenous ATP applications. Each protocol was replicated in at least 4-5 different oocytes attained from separate oocyte batches from separate *X. laevis* frogs. The animals were carefully handled before, during, and after surgery; in accordance with the principles of the Helsinki declaration on animal welfare and our Faculty and University ethical procedures. All protocols regarding oocyte microinjections were approved by the Universidad de Santiago Ethical Committee as well as by the local Faculty Ethical Committee (protocol code 471-2017). Injected oocytes were stored at 12°C for 36–48 h in Barth's incubation solution containing (mM): 88 NaCl; 1 KCl; 2.4 NaHCO_3_; 10 HEPES; 0.82 MgSO_4_; 0.33 Ca(NO_3_)_2_; 0.91 CaCl_2_; pH 7.5 supplemented with 10 IU/L penicillin/10 mg streptomycin, and 2 mM pyruvate. On the testing day, oocytes were clamped at −70 mV using the two-electrode voltage-clamp configuration with an OC-725C clamper (Warner Instruments Corp., Hamden, CT, USA). The 1 μM ATP-gated currents were recorded following regular 10 s nucleotide applications, which were repeated at regular 10-min intervals. Recovery of control ATP-gated currents was always assessed. ATP, Zn(II) and the IVM stock solution in DMSO were dissolved in Barth's media and perfused to the oocyte using a pump operating at constant flow (2 mL/min). Control DMSO experiments were performed to rule out a possible solvent effect. The procedure for oocyte dissection and P2XR expression followed the Acuña-Castillo et al. (2007) or Coddou et al. (2007) detailed protocols.

#### Modulator action of IVM and Zn(II) in wt P2XRs

To examine the IVM specificity as a P2XR modulator, oocytes expressing the rat P2X4, P2X2, or P2X7R were tested by application of 3 μM ATP preincubated with 3 μM IVM for 3 min, following the Silberberg et al. (2007) protocol. IVM was dissolved in DMSO; the final dilution was less than 0.1%, a vehicle concentration that did not affect ATP-evoked currents of control oocytes. In all protocols, full reversal of the IVM effect was mandatory prior to assessing a following IVM concentration. Recovery of the basal ATP-gated currents was assessed comparing the magnitude of ATP-gated currents every 8–10 min. The same protocol was followed using oocytes administered with P2X2R and P2X7Rs. The results are expressed as the fold-increase in the ATP-evoked currents.

To assess the nature of the metal modulator action, 10 μM Zn(II) was always pre-applied for 1 min before 1 μM ATP applications. Reversal of the metal modulator effect was always checked by comparing the ATP-evoked currents in the same oocyte after prolonged metal washout. The results are expressed as the fold-increase in the ATP-evoked currents. To confirm the results observed in oocytes, identical protocols were performed with HEK 293 cells expressing the wt P2X4R. To this end, 3 μM IVM or 10 μM Zn(II) pre-applications were performed for 3 min or 1 min prior to the 1 μM ATP application.

#### Joint application of IVM and Zn(II) in wt P2XRs

The same oocyte was used to assess the action of both modulators applied simultaneously: 3 μM IVM was first applied, followed 2 min later by a 1 min pre-application of 10 μM Zn(II). One minute later, oocytes were challenged with 1 μM ATP. As controls, the same oocytes were previously tested separately with either 3 μM IVM or 10 μM Zn(II). Experiments where one of the modulators showed a statistically weak response were discarded, reducing experimental variability (2/48 cases). The results are expressed as the fold-increase of the 1 μM ATP-evoked currents. Oocyte findings were confirmed in HEK293 cells expressing the wt P2X4R.

#### Experiments with P2X4R mutants and P2X4/2R chimera

Similar protocols as described previously were performed in oocytes injected with the following P2X4R mutants: C132A, C126A or the P2X4/2R chimera. C126A and C132A mutated P2X4Rs and chimeric P2X4/2R. Mutants were synthetized as reported by Coddou et al. (2007); the P2X4/2R chimera contained the extracellular domain of the P2X4R but the membrane and intracellular domains of the P2X2R (He et al., 2003). This chimera was a personal contribution of Dr. S. Stojilkovic (National Institute of Health, Bethesda, USA) for this particular study. We next examined the relevance of the Zn(II) and IVM potentiation in oocytes injected with either C126A or C132A P2X4R mutants. For this purpose, we recorded the currents gated by 1 μM ATP, and ATP in the presence of either 10 μM Zn(II) or 3 μM IVM, or both modulators co-applied. Reversibility of the two modulator s' effects was mandatory prior to continuing with the protocol. All experiments tested the action of both modulators on the same oocyte.

Chimeric receptors: Protocols first examined the magnitude of the 3 μM ATP-gated currents in oocytes and HEK cells, respectively, in the presence of either 10 μM Zn(II) or 3 μM IVM alone. Next, we assessed the ATP-gated currents exposed to both modulators applied simultaneously in the same cell. These protocols were examined in oocytes as well as in HEK cells.

#### HEK 293 cell transfection and electrophysiological recordings of P2X4R wt and chimeras

HEK 293 cells were stably transfected with 1 μg cDNA for the wt P2X4R or the P2X4/2R chimera. Cells were grown in DMEM media to 80% confluence on 35-mm culture dishes and incubated with the cDNA mixed with 10 μL of Lipofectamine 2000 in 1 mL of serum freemedia (Opti-MEM, Invitrogen, Carlsbad, California, USA). The P2X positive colonies were selected with G 418 during at least 2 weeks.

Whole-cell ATP-gated currents were recorded from single HEK 293 cells using an Axopatch 200B amplifier (Axon Instruments, Foster City, CA). Patch pipettes (2–4 megohm) were filled as follows (mM): 150 CsCl, 10 tetraethylammonium chloride, 10 EGTA, 10 HEPES, pH 7.3, and 275–285 mOsm; pH was adjusted with CsOH (Coddou et al., 2003a). The recording chamber was perfused with a solution containing (mM): 150 NaCl, 1 CaCl_2_, 1 MgCl_2_, 10 glucose, and 10 HEPES. Only cells with membrane potential more negative than −55 mV, access resistance < 10 MΩ, and input resistance >00 MΩ were assessed. ATP-gated currents were assessed recording at a holding potential of −80 mV. Responses were digitized at a frequency of 10 kHz and analyzed using the pCLAMP 8 from Axon Instruments (Foster City, CA). All protocols were conducted at room temperature (22–25°C).

### Drugs and chemicals

ATP as the trisodium salt, zinc chloride, tetraethylammonium chloride, EGTA, HEPES, and penicillin-streptomycin were purchased from Sigma Chemicals (St. Louis, MO, USA). Lipofectamine 2000 was purchased from Invitrogen. G-418 sulfate was obtained from Calbiochem (San Diego, CA, USA). The salts used to prepare the incubation media were obtained from Sigma-Aldrich or Merck (Darmstadt, Germany). Triple-distilled water with minimal electro conductivity was produced locally; its trace metal contamination was less than 0.1 μM.

### Statistical data analysis and expression of electrophysiological results

Non-parametric analyses were performed as appropriate (Kruskal-Wallis Friedman or Mann-Whitney tests); we previously determined the convenience of nonparametric analysis procedures (Theodorson-Norheim, 1987) for statistical evaluations of oocyte studies. Significance was set at *p* < 0.05, (^*^).

ATP-gated currents are always expressed as the fold-increase in the ATP-evoked response (normalized current) when comparing the control ATP-gated currents with that elicited in the same oocyte or HEK cell, in the presence of either Zn(II), IVM, or both modulators applied jointly.

## Results

### IVM selectivity as an allosteric P2X4R modulator

Based on the rat P2X4R model, the TM residues, previously proposed by Popova et al. (2013) to interact with IVM, were aligned with those present in the P2X2R and P2X7R to ascertain the role of IVM allosteric modulation. While Y42 is conserved in these three P2XRs, W46 was only found in the P2X4R. In addition, two single residue replacements were observed among the P2XRs. W46 in P2X4R was replaced by Y46 (P2X2R) and F46 (P2X7R), while W50 in P2X4R was replaced by V50 (P2X2R) and S50 (P2X7R) (Table 1). Moreover, we determined the free energies of transfer from water to ethanol (Nozaki and Tanford, 1971; von Heijne and Blomberg, 1979), and we also examined the hydrophobicity score of amino acid side chains/backbone localized in the extracellular area immediately close to the TM domain. These calculations determined the hydrophobic environment related to the IVM putative TM domain allosteric binding site. In agreement with this proposal, P2X4R showed the highest hydrophobic score (−33.75 kcal/mol) followed by P2X7R (−40.55 kcal/mol) and the P2X2R (−53.8 kcal/mol), data confirming the physicochemical characteristics favoring IVM access to its binding site. A graphical representation of this data is given in Supplementary Figure 1.

**Table 1:** Alignment of transmembrane P2X4R, P2X2R, and P2X7R rat sequences.

	**Receptor**	**Sequence**
TM1		42 46 50
	ratP2X4R	28VGLMNRAVQLLILAYVIGWVFVWEKGY54
	ratP2X2R	28LGFVHRMVQLLILLYFVWYVFIVQKSY54
	ratP2X7R	28YGTIKWILHMTVFSYVS-FALMSDKLY53
TM2		336 341 349
	ratP2X4R	331DIIPTMINVGSGLALLGVATVLCDVIVL358
	ratP2X2R	331SLIPTIINLATALTSIGVGSFLCDWILL358
	ratP2X7R	331DIIQLVVYIGSTLSYFGLATVCIDLIIN358

*Key amino acids previously reported to participate in P2X4R IVM modulation are written in red, showing the coincidence with the present residues obtained by molecular docking (gray boxes). The corresponding residues in P2X2R and P2X7R, enumerated from the alignment with the P2XR sequence, are shown in orange*.

**Figure 1:**
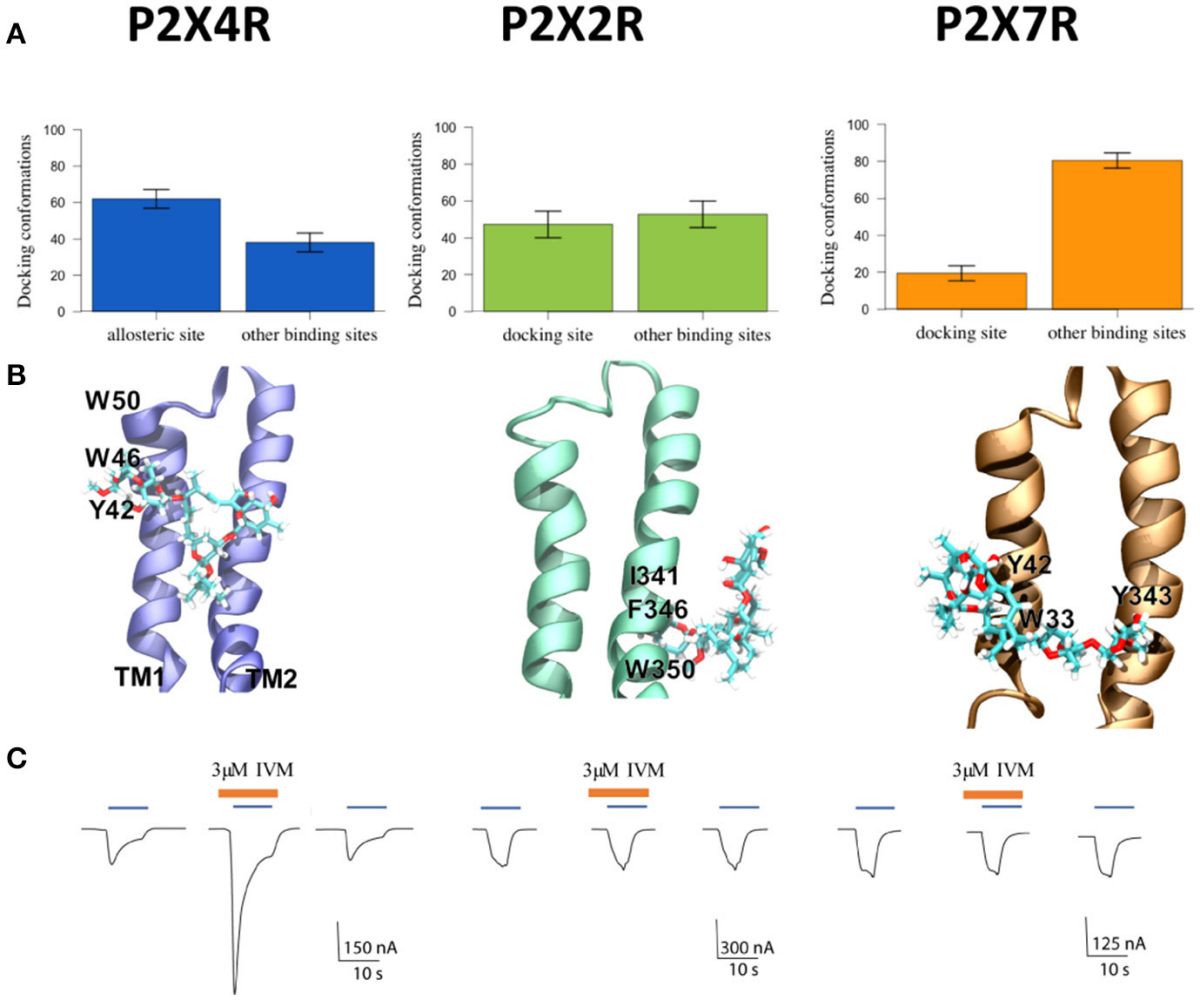
Characterization of P2XR-IVM binding specificity by *in silico* and electrophysiological approaches. **(A)** Number of conformations obtained by molecular docking of IVM to rat P2X4R, P2X2R, and P2X7R. **(B)** Representative best energy conformation of IVM docking with the three P2XRs studied; lowest IVM P2X2R binding energy to other sites not analogous to the allosteric site in the P2X4R. IVM binding showed interaction with I341, F346, and W350 residues. In the case of P2X7R, IVM docking interacted with Y42, W33, and Y343. **(C)** Representative recordings of 1 μM ATP-gated currents in *Xenopus laevis* oocytes pretreated with 3 μM IVM (brown line) for 3 min prior to 1 μM ATP addition (blue line) in separate oocytes, each expressing P2X4R, or P2X2R, or P2X7R, respectively. Calibration scales are different for each oocyte-evoked currents during 10 s. ^*^p < 0.05 between the allosteric or docking and other binding sites. #*p* < 0.05 between the allosteric site in the P2X4R and putative docking sites in the P2X2R or the P2X7R.

Following the P2XRs alignment, the molecular docking studies at the allosteric binding site provided the binding probability to this site versus other non-related sites in the close P2XR vicinity. Docking analysis revealed that IVM has a 62% higher probability of binding to the described P2X4R allosteric site (*p* < 0.05) compared to other sites in the TM region. Moreover, the probability of IVM binding to a similar site in the P2X2R was significantly lower (48%) and even less in the P2X7R (18%). Furthermore, the probability of IVM binding to other sites was 52 and 82% for the P2X2R and P2X7R respectively (Figure 1A). Based on these findings, graphical representations of the preferred binding mode of IVM to the three P2XRs examined are shown in Figure 1B. The docking of IVM to P2X4R involves π-π stacking interactions with Y42 and W46 in the TM1 domain, plus hydrogen bonding to S341 in the TM2 domain. Moreover, W46 and W50 clearly show a π-π interaction which influences the aromatic ring orientation, a finding not observed in P2X2R or P2X7R at the analogous binding place (Table 1, Figure 1B). The calculated IVM binding energy for its P2X4R binding site was −6.85 kcal/mol.

Consistent with bioinformatics, electrophysiological recordings of ATP-gated currents in P2XRs showed that only the P2X4R was positively modulated by IVM (Figure 1C). Interestingly, although a 48% probability of IVM binding to the P2X2R putative allosteric binding site was determined and the hydrophobic environment seems compatible with an IVM chemical nature (Table 1, Supplementary Figure 1), it was not enough to trigger an ATP-gated current, reflecting the IVM selectivity for the P2X4R. Altogether this set of evidence leads us to firmly propose the structural bases for IVM P2X4R selectivity.

**Figure 2:**
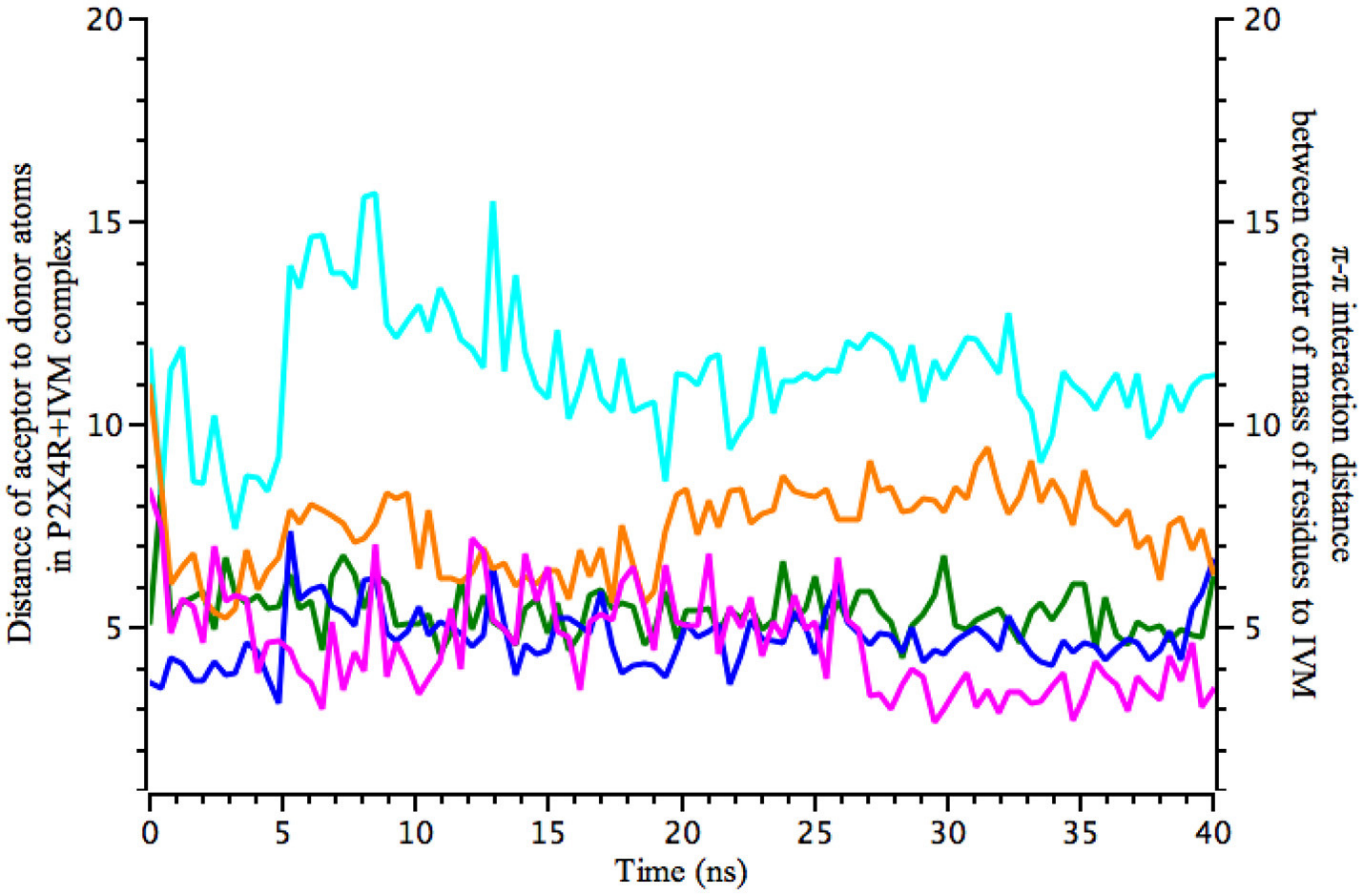
Molecular dynamics calculations show the distance of IVM interaction with P2X4R. Calculated 40 ns MD simulations of IVM interaction with the holo P2X4R. Left y-axis plots the distance (in Å) between acceptor to donor atoms (hydrogen bonds), calculated between O1 of S341 (magenta) with O4 of IVM, and O1 of D331 (orange) with O12 of IVM. Right y-axis refers to possible π-π stacking interactions between W50 (blue sky), W46 (blue), and Y42 (green), calculated between center of mass of the aromatic residues and IVM atoms.

MD simulations revealed that π-π stacking interactions were maintained at the expected 5 Å distance throughout the simulation time, and hydrogen bonding of IVM to D331 oscillates between 2.5 and 5 Å, indicative of dynamic hydrogen bonding fluctuations. Calculations are also consistent with a relative conserved distance of 3–5 Å between IVM and S341, which at 28 ns shows an abrupt reduction of the distance to approximate 2.5 Å, stabilizing the hydrogen bond with IVM (Figure 2).

### The Zn(II) modulator site

**Figure 3:**
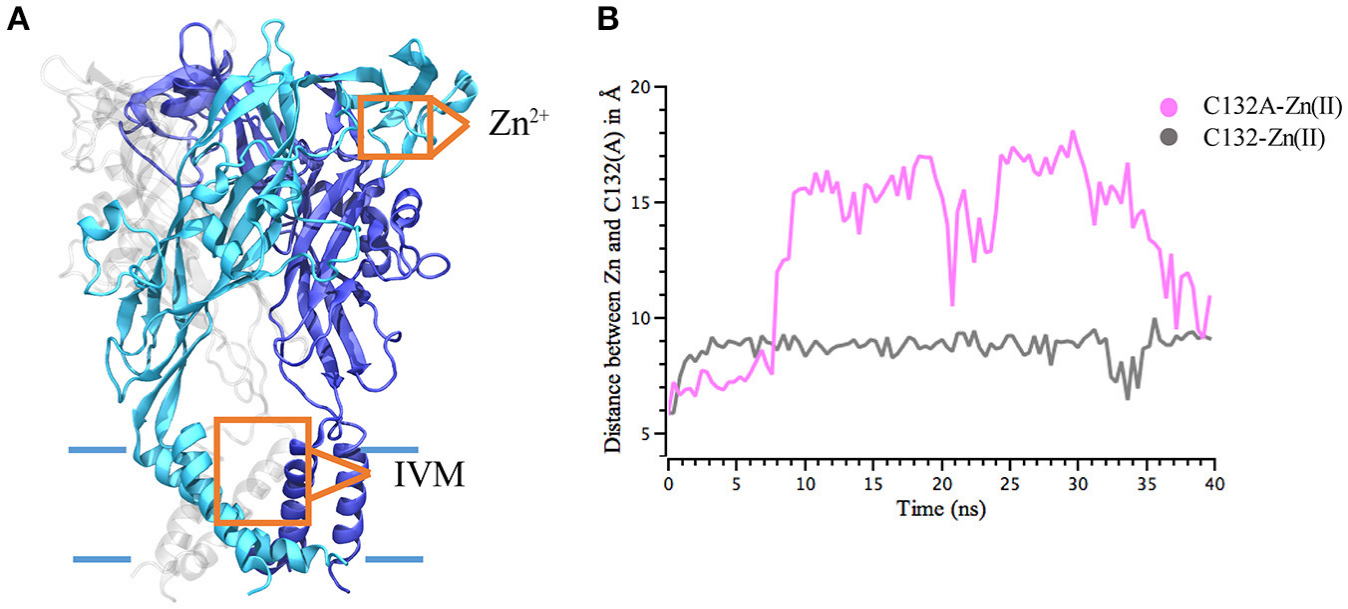
Interaction distance between Zn(II) and P2X4R calculated through molecular dynamics simulations. Calculated 40 ns MD simulations of Zn(II) interaction with the P2X4R holo. **(A)** Model of P2X4R showing the putative Zn(II) site in the extracellular domain and IVM binding site in the TM region. **(B)** Distance between Zn(II) and S atom of C132wt P2X4R (gray) or Cβ of the C132A mutated P2X4R (pink).

IVM and Zn(II) interact on separate binding sites (Figure 3A) and elicit biological responses. C132 in the P2X4R extracellular domain was previously identified as a key residue involved in Zn(II) modulation (Coddou et al., 2007). To further characterize the role of C132 in a wt and the C132A P2X4R mutant, we studied the stability of P2X4R Zn(II) based on the distance between C132 and the Zn atom. MD simulations showed that a single Zn(II) positioned at 5 Å from the wt P2X4R C132 sulfur atom (SS3), maintained its position at a distance of less 8–10 Å during 40 ns. In the case of the C132A mutant, Zn(II) was also placed at 5 Å from an alanine C_β_. Simulations show that the carbon distance was maintained only for the first 6 ns (Figure 3B); thereafter the Zn(II) suffered a sudden separation reaching a distance of 16–18 Å that was maintained for the next 35 ns. As negative control, a Zn(II) ion positioned in the extracellular domain at 8 Å from C132 sulfur showed a steady distancing from the P2X4R, reaching distances greater than 80 Å after 40 ns.

### Pore radius and lateral fenestration dimensions of P2XRs bound to IVM, to Zn(II), and to both modulators

In agreement with the findings that IVM and Zn(II) interact at separate binding sites and elicit differential biological responses, we searched for structural changes in the pore size. To this end, a HOLE trajectory analysis of P2X4R apo, and holo systems with one or both modulators bound were performed.

**Figure 4:**
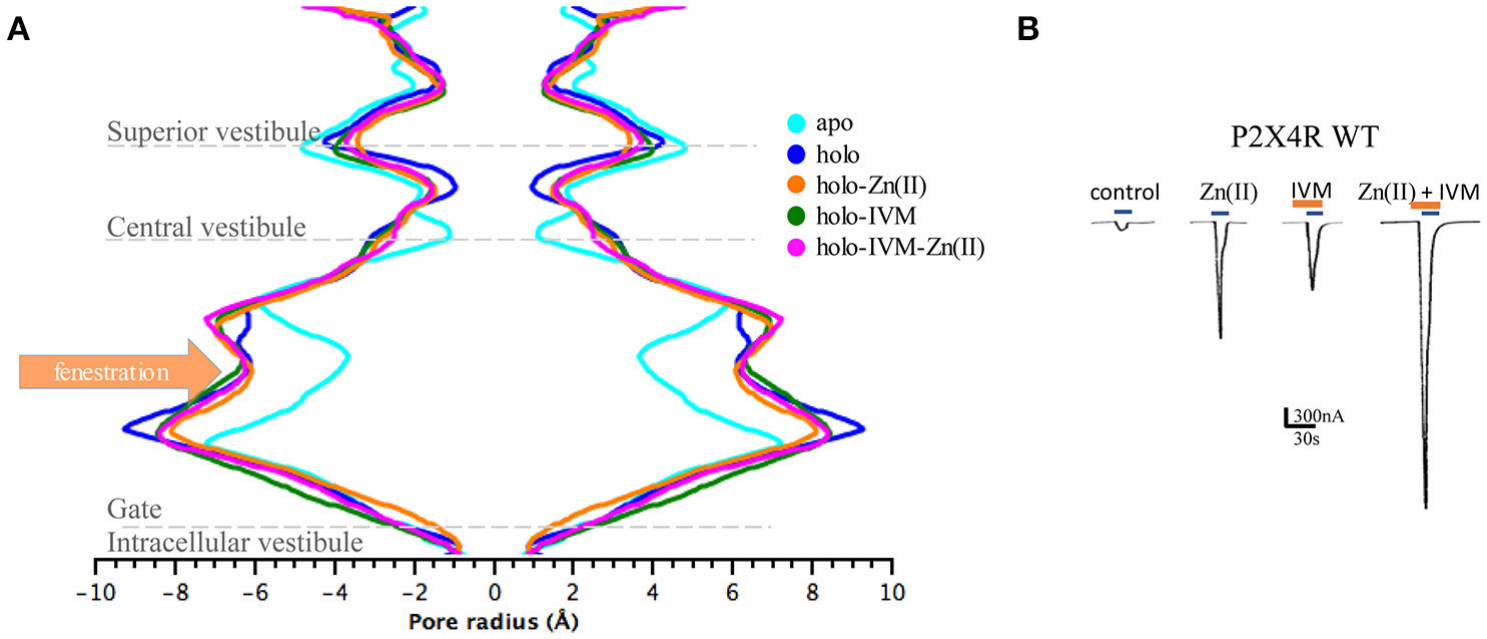
Allosteric modulators induced changes in the pore radius of the extracellular P2X4R vestibule in different states of the P2X4R. **(A)** Pore dimensions obtained during the last 10 ns MD simulations of the P2X4R in apo or holo state, holo with Zn(II) or with IVM, and the holo P2X4R with both IVM and Zn(II), along the vestibular portions of the P2X4R. **(B)** Representative tracings obtained in a single oocyte expressing the wt P2X4R following 1 μM ATP-evoked currents in the absence of modulators, and following incubation with either 10 μM Zn(II), 3 μM IVM, or the joint application of IVM plus Zn(II). In all cases 1 μM ATP-evoked currents were recorded (blue rectangles), while IVM applications are shown in brown rectangles.

**Figure 5:**
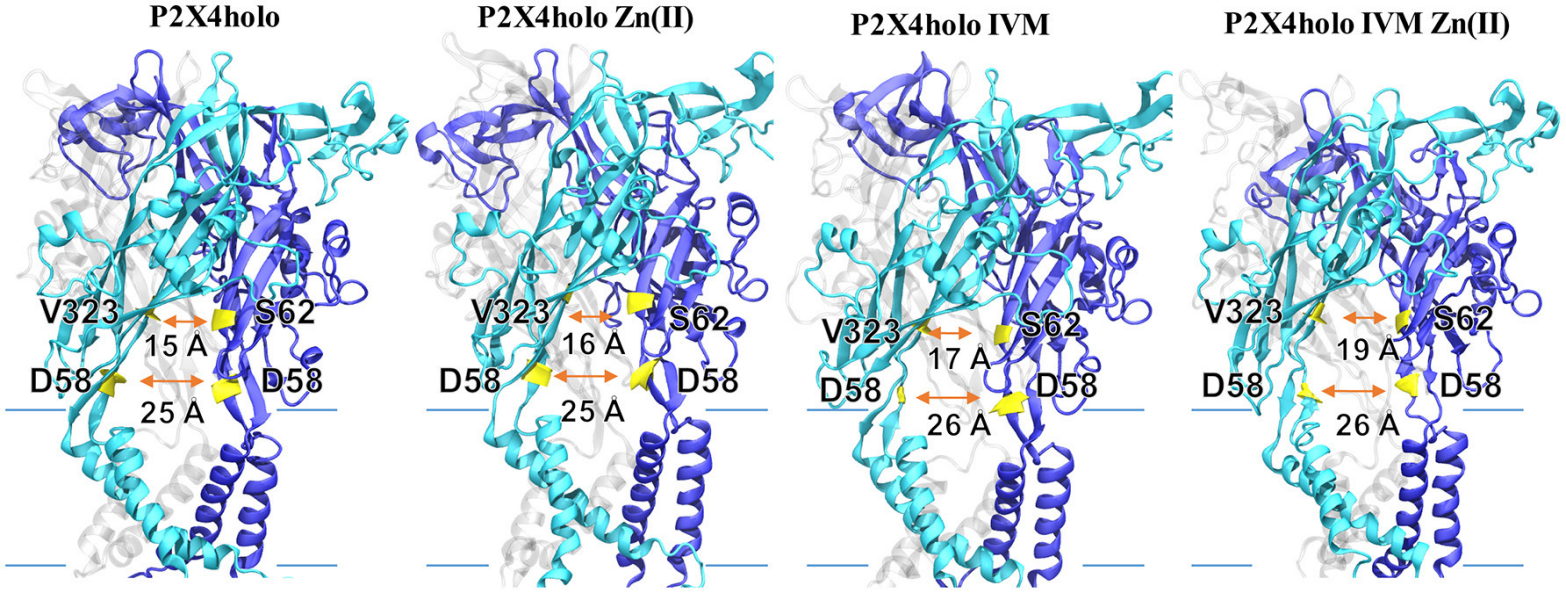
Lateral fenestration dimension of P2X4R in different states. Models represent the P2X4R holo state with either Zn(II), IVM, or both bound. Lateral fenestration size was calculated in the apo as well as the holo state, holo in the presence of Zn(II) or IVM, or holo plus IVM and Zn(II). Backbone distance between D58 with its corresponding D58 in the adjacent P2X4R subunit was considered. Likewise, the backbone distances between V323 with S62 in the adjacent subunit were determined. Lateral fenestration dimensions are expressed in Å.

P2X4R in the presence of ATP plus Zn(II) and IVM resulted in an enlargement of the upper part of the extracellular vestibule (7.97 ± 0.01 Å, *p* < 0.05), compared to the holo P2X4R which is 6.03 ± 0.3 Å (Table 2, Figure 4A). When the P2X4R is activated by ATP plus Zn(II) or ATP plus IVM, the calculated size of the upper vestibular portion of the pore size was significantly smaller, 6.93 ± 0.2 Å and 7.03 ± 0.3 Å, respectively (*p* < 0.05). Altogether, these results support a metal-induced enlargement of the pore radius and lateral fenestration by the allosteric modulator permitting a larger intracellular ionic flux. This fact is supported by electrophysiological findings, ATP-gated currents were increased 15.1 ± 3.7-fold (*n* = 4) by 10 μM Zn(II) and 6.9 ± 1.2 fold (*n* = 5) by 3 μM IVM. When both modulators were applied jointly (IVM pre-applied for 3 min and Zn(II) for 1 min prior to the 1 μM ATP challenge), a 30.8 ± 5.5-fold increase of the ATP-gated current was observed *(p* < 0.01 compared to either modulator alone). Representative recordings are shown in Figure 4B. Regarding the P2X4R holo system, the lateral fenestration at the membrane interphase was increased 4 Å after binding of both modulators, while Zn(II) and IVM applied separately only triggered an increase of 1 Å and 2 Å, respectively (Figure 5). Similarly, concomitant experiments on P2X4R transfected HEK cells, 10 μM Zn(II) alone doubled (*n* = 9) and IVM tripled the ATP-evoked currents (*n* = 6); the joint application of these modulators resulted in an almost 4-fold larger current (*n* = 6, data not shown). Taken together, both *in silico* and electrophysiological results are consistent with an additive effect rather than a strong synergic allosteric interaction.

**Table 2:** Radius of P2X4R pore in the apo, holo, and holo plus allosteric modulators; (values are expressed as the mean ±standard deviation; values in Å).

**P2X4R compartment**	**P2X4 apo**	**P2X4 holo**	**P2X4 holo IVM**	**P2X4 holo Zn(II)**	**P2X4 holo IVM Zn(II)**
Upper vestibule	2.10 ± 0.19	1.83 ± 0.21	1.24 ± 0.28	1.34 ± 0.22	1.28 ± 0.20
Upper extracellular vestibule	5.95 ± 0.09	6.03 ± 0.30^*^	7.03 ± 0.26^*^	6.93 ± 0.15^*^	7.96 ± 0.01^*^
Extracellular vestibule	7.21 ± 0.23	9.27 ± 0.27^*^	8.45 ± 0.59	8.11 ± 0.58	8.40 ± 0.64
Intracellular vestibule	0.28 ± 0.07^*^	0.88 ± 0.1^*^	0.97 ± 0.28	0.86 ± 0.34	0.80 ± 0.28

Radius values were calculated following HOLE program analysis during the last 10 ns of the MD simulations lasting 30–40 ns (or 40–50 ns when allosteric modulators were present). The minimum radius of the upper vestibule was calculated, the maximum radius of the extracellular vestibule was also calculated as the minimum value of the intracellular vestibule (

* *p < 0.05 compared to P2X4R apo state)*.

### Non-bonded energy calculations of the different P2X4R states with and without ATP and modulators

**Figure 6:**
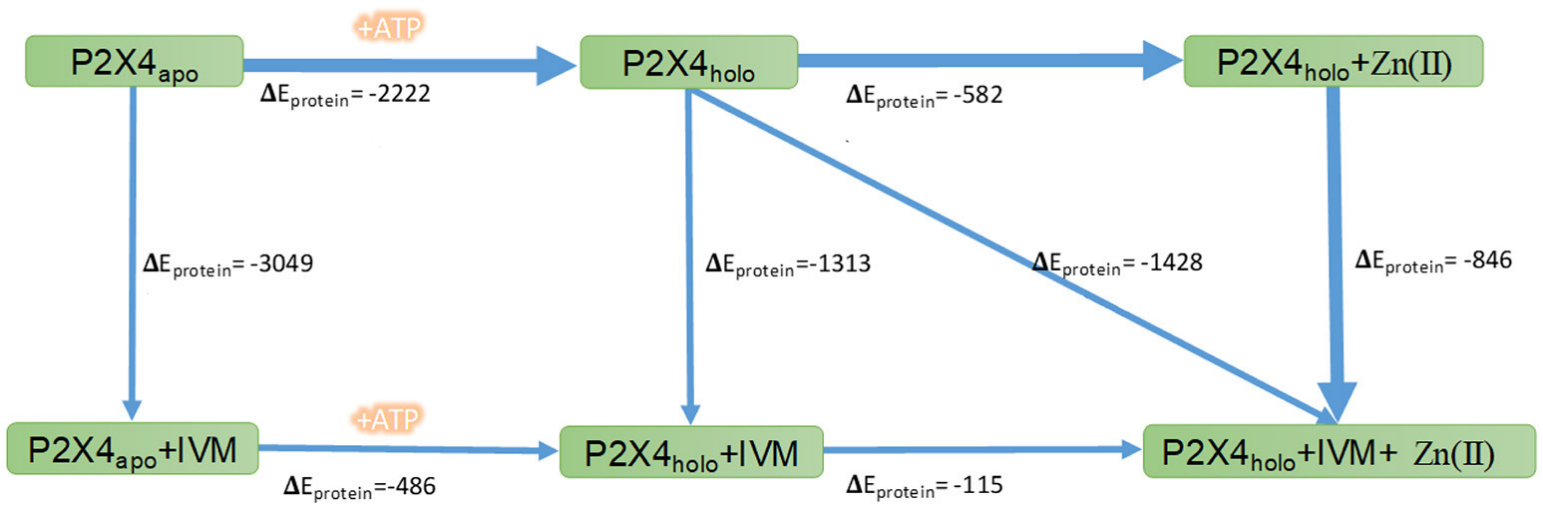
Non-bonded energy determination of the P2X4R in different states. Numbers indicate the changes in non-bonded energies, expressed as kcal/mol, of the whole wtP2X4R (ΔE_protein_) in the different conformational complexes of the P2X4R. The rectangles indicate P2X4R states in the apo or holo state alone or following allosteric modulator binding. The arrows refer to the transition between the different states. The thicker blue lines indicate the favored P2X4R transitions after allosteric modulator binding.

We calculated energy changes elicited by the P2X4R transition steps from apo to its holo state as well as IVM and/or Zn(II) binding complexes, and the difference in non-binding energies elicited by ATP and modulator binding was examined. The apo to holo state transition is favored by the energy change of −2222 kcal/mol. Likewise, the sequence of IVM binding to the holo P2X4R is energetically more favorable compared to the apo P2X4R binding (−3383 vs. −3090 kcal/mol, Figure 6). In addition, our calculations indicated that Zn(II) causes transitions requiring more energy than IVM, leading to a more favorable and stable P2X4R conformation in the presence of IVM. Based on these calculations, we inferred that Zn(II), due to its lower molecular weight and charge, has fewer degrees of freedom compared to IVM, leading to an increased P2X4R dynamic movement. Energy calculations for the different transitions are consistent with the following sequence of steps accounting for P2X4R modulation: 1. Apo to holo transition; 2. Holo P2X4R IVM complex; 3. Holo P2X4R Zn(II) IVM. These favorable P2X4R transitions are shown by the thicker black arrows represented in Figure 6.

### Studies with the P2X4R C132A mutant and a P2X4/2R chimera

**Figure 7:**
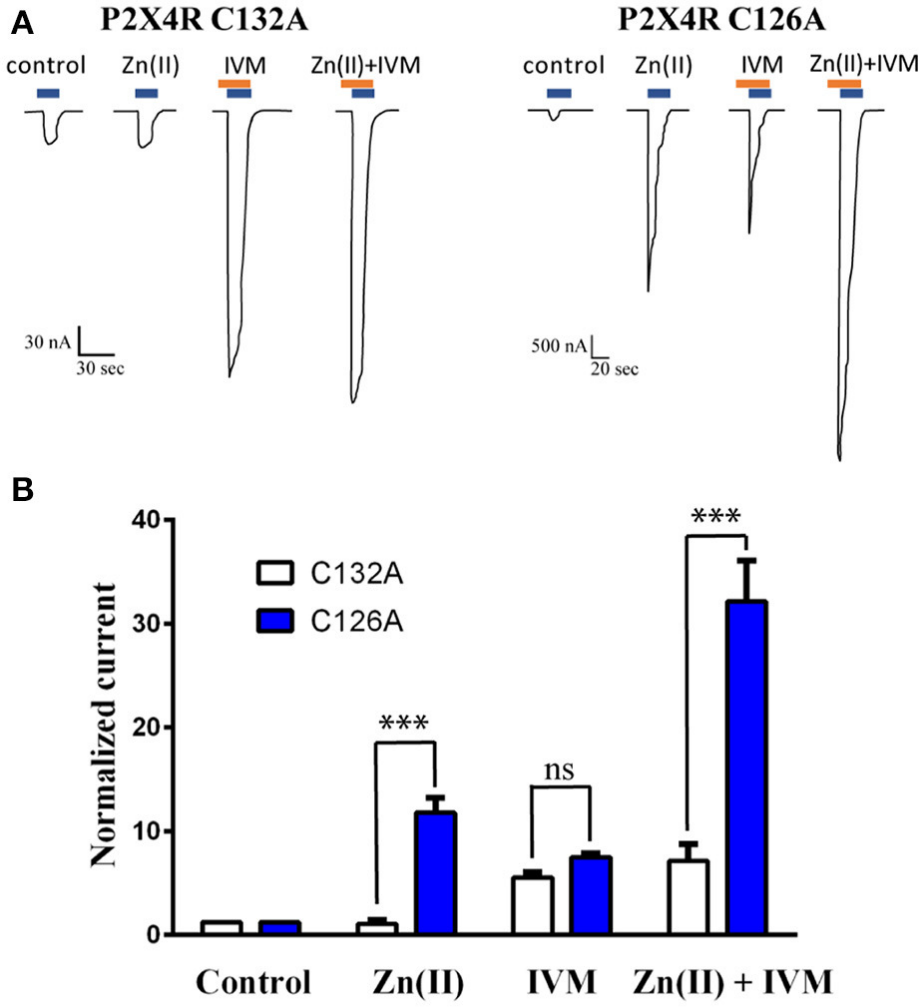
Loss of Zn(II) modulator activity in the C132A P2X4R mutant but not the C126A mutant. **(A)** Representative recordings of 1 μM ATP-gated currents in two P2X4R mutants (C132A and C126A) in the absence or presence of 10 μM Zn(II), 3 μM IVM and joint application of Zn(II) plus IVM. ATP and IVM applications are represented by blue and brown rectangles, respectively. Each set of recordings is derived from a single oocyte expressing either the C132A or the C126A mutants. **(B)** Statistical analyses of a set of 5 separate oocytes expressing the C132A mutant (open bars) and 4 oocytes expressing the C126A mutant (closed bars) (***p < 0.001); ns (not statistically significant).

Based on docking and MD simulations we predicted that the P2X4R C132A mutant, should be insensitive to the allosteric action of Zn(II), while it should preserve the modulator action of IVM. As an electrophysiological control for this protocol, we examined the P2X4R-C126A mutant, since a structurally equivalent mutation, P2X4R-C132A, is located in close vicinity. Parallel studies proved that the Zn(II)-induced potentiation of the receptor activity was retained (Coddou et al., 2007). The data confirmed that the C132A mutant is not sensitive to the modulator action of Zn(II), since it failed to modify the 1 μM ATP-gated current compared to the wt (Figure 7A). In contrast, Zn(II) applications to the C126A mutant increased 11.6 ± 1.5-fold the ATP-gated current (Figure 7B). Moreover, and in support of our contention, 3 μM IVM increased the ATP-evoked currents 4.9 ± 0.7–fold (*n* = 10) and 7.3 ± 0.5-fold (*n* = 8) in the C132A and C126A mutants, respectively, values which are not statistically significant between them. The joint application of Zn(II) plus IVM resulted in a 7.1 ± 1.7-fold increase of the ATP-evoked currents (*n* = 9, Figure 7B), a value not statistically different from that elicited by IVM alone, while in the C126A mutant the increase in the ATP-gated current was 31.9 ± 4.1-fold (Figure 7B), confirming that both modulators act at separate and apparently independent allosteric modulator sites and that the joint action of both modulators elicited additive effects.

As a proof of concept, we took advantage of a P2X4/2R chimera constituted by the extracellular P2X4R domain and the TM plus intracellular P2X2R domains. Based on the *in silico* studies, we again predicted, that this construct should be insensitive to IVM. Electrophysiological findings showed that 10 μM Zn(II) application increased 2.7 ± 0.6-fold (*n* = 5, *p*-value < 0.001) the 1 μM ATP-gated current, a value which is significantly less than that observed in the wt P2X4R (Figure 4B). Prominently, and as anticipated, the chimera was insensitive to 3 μM IVM (1.1 ± 0.1-fold increase, *n* = 5); the joint application of both IVM plus Zn(II) did not increase further the magnitude of the ATP-gated currents (2.7 ± 0.9-fold, *n* = 3, *p* < 0.05). These results were confirmed in HEK cells expressing the P2X4/2R chimera; almost identical values were attained for IVM and the joint application of IVM plus Zn(II) (1.55 ± 0.1 (*n* = 3) and 1.4 ± 0.2 (*n* = 4), respectively. Although experimental values of the currents recorded from HEK cells were slightly reduced in magnitude, the results agree with a general picture compatible with the proposed hypothesis that guided this study.

## Discussion

The present set of results revealed the advantages of using complementary approaches to study the mechanisms of action of P2X4R allosteric modulators. MD simulations allowed the visualization of the most likely initial conformational changes occurring in the receptor by allosteric interactions, whose interpretation benefits from prior pharmacological investigations using site-directed mutagenesis on P2XRs (Coddou et al., 2007), and in particular benefits from the results with the P2X4/2R chimera in the present study.

Our data strongly suggest that the IVM and Zn(II) allosteric binding sites are distinct and operate by separate mechanisms. The joint application of IVM plus Zn(II), each acting at its specific allosteric site, caused additive rather than synergic effects. Moreover, bioinformatics concurred to identify the Zn(II) allosteric site, which is localized in the extracellular receptor domain, at a site not totally identified as yet, but in the near vicinity of C132 comprising the SS3 receptor bond. In contrast, the IVM site, as previously proposed, is restricted to the P2X4R TM domain. This paper provides precise docking studies identifying details of the IVM binding site characteristics, in agreement with the hypothesis that IVM is inserted between two neighboring TM domain of the P2X4R subunits and restricts molecular rearrangement in the TM domains involved in channel gating. A similar finding was reported for the Cys-loop receptor family of ligand-gated ion channels (Hibbs and Gouaux, 2011), showing functional commonalities of allosteric regulation among other ionic channel families.

To address and compare IVM selectivity for the P2X4R, we conducted parallel calculations on the P2X2R or the P2X7Rs. Based on the sequence alignment of these P2XRs, plus the IVM docking studies, we concluded that TM1 plays a critical role in the interaction, highlighting hydrophobic and stacking interactions with Y42 and W46 plus a minor role of W50. These interactions assisted the positioning of IVM at this site, creating a non-polar environment for the drug entry to this binding pocket essential for IVM activity (Jelínková et al., 2006). The interaction is further stabilized by hydrogen bonding to S341 of the adjacent subunit and other residues with less propensity for hydrogen bonding, a bond that occurs relatively late in time compared to the hydrophobic forces. The immediate environment of this P2X4R area, in the vicinity of the lateral fenestration (Supplementary Figure 1), is relatively hydrophobic in nature, a finding supported by IVM hydrophobicity (log P = 5.83, Viswanadhan et al., 1989). Additionally, the P2X4/2R chimera construct was insensitive to IVM, a result that correlates with the in silico studies. Moreover, mutation of four amino acids of the lateral fenestration, Y195, F198, F200, and F330, altered the ability of IVM to sensitize P2X4R to ATP, revealing their contribution to the channel function and deactivation effect (Gao et al., 2015). Although docking studies revealed a 62% probability of IVM binding at this specific site, we are aware that 38% of non-specific interactions are also formed. This binding dominance to a preferred site is in marked contrast with the P2X7R docking results, where non-specific binding is close to 82%. Although the binding probability is almost half in the P2X2R, electrophysiological results consistently demonstrated that the P2X2R is not IVM-modulated. We infer that neither IVM binding to TM1 aromatic residues nor the possible hydrogen bonding to P2X2R or P2X7R TM2 residues confer enough stability compatible with eliciting the correct conformational change related to the allosteric modulation.

Considering the trimer nature of P2X4R, it has not escaped our interest to determine whether three IVM molecules bind to the receptor. It was our working hypothesis that at a given IVM concentration range, all three allosteric receptor sites should be occupied. To this end, longer dynamics are critical to investigate whether all three IVM molecules reach and stabilize the P2X4R at the same time or whether this occurs gradually, suggesting binding cooperativity, where the first IVM binding facilitates the subsequent binding of the next IVM molecule. This proposal is supported by the Markov model presented by Zemkova et al. (2015). A similar argument could be argued for the binding of Zn(II) to its allosteric site in the extracellular P2X4R domain. In the case of the metal, this is even more attractive, since its allosteric site is distant from the pore. Energy determinations indicated that Zn(II), in contrast to IVM, evidenced a larger standard deviation of the non-bonded electrostatic energies derived from the MD studies. The latter might be indicative that Zn(II) elicited a favorable electrostatic component in the ionic flux to intracellular compartment (V. Latapiat et al., data not shown). Taken together, these findings validate the result obtained with the C132A P2X4R mutant insensitive to the allosteric action of Zn(II), but that preserve the modulating action by IVM. This result is different from those observed with C126A, confirming the predominant role of C132 in the Zn(II) allosterism. In addition, it also supports that the joint action of both modulators is additive, as will be discussed in the next.

An exciting issue analyzed in this investigation refers to the pore size widening by allosteric modulators, an enterprise worth bioinformatics analysis. Present studies consistently showed that ATP opened the pore and lateral fenestrations, but not the intracellular vestibule, which in the case of P2XRs is directly associated with the ion flux to a larger extent than the central pore (Kawate et al., 2009). Both IVM and Zn(II) enlarged the upper region of the extracellular vestibule, but only within a 1 Å width (Table 2), an amount which was increased to 2 Å by the joint application of both modulators, an issue that was further supported by an additional experiment. A classical biophysical approach to explore pore size consists in replacing sodium by N-methyl D glucamine, a charged molecule with a radius 4–5 times larger than Na^+^. Preliminary experiments showed that the sodium replacement by the N-methyl D glucamine abolished the ATP-induced currents in oocytes injected with wt P2X4R, a reversible effect since ATP-gated currents were 100% recovered after sodium substitution. Moreover, addition of 3 μM IVM plus 10 μM Zn(II) did not elicit an ATP-gated current (F. Peralta, unpublished observations). This result confirms the bioinformatics calculations indicating that the pore opening did not permit pore enlargement to 4.5 Å radius, which is incompatible with N-methyl D glucamine diffusion through the ATP channel.

A most enlightening aspect of the combined bioinformatics plus electrophysiological assays confronts the non-bonded energy calculations of the changes occurring by allosteric modulators bound to apo or holo states of the P2X4R. Apo to holo transition is necessary to cause lateral fenestration opening that allows the entrance of IVM to its binding site. Once the holo state is present, the calculations indicate that the most favorable transition energy corresponds to the IVM binding, which is in agreement with the electrophysiological procedure testing the joint modulator application, where IVM is pre-incubated with Zn(II). This could indicate a much greater influence of the hydrophobic energy of the IVM binding the TM region rather than a more electrostatic role of the Zn(II) binding site. The electrophysiological experiments showed that IVM needs to be pre-incubated 3 min prior to ATP addition, a time needed for IVM to reach the vicinity of the lateral fenestration. The concentration response curves of P2X4R revealed that not only 10 and 30 s pretreatments with IVM increased ATP sensitivity, but they also increased the maximum current amplitude evoked by ATP (Mackay et al., 2017). In agreement with this, molecular docking of IVM to the apo P2X4R showed less than 5% binding to its allosteric site. On the other hand, Zn(II) as a modulator does not require pre-incubation, probably due to the fact that its binding site is easily accessible from the extracellular space and does not require the holo P2X4R state. Since the intracellular vestibular size was not significantly an increased by these modulators, the increased ion currents observed experimentally imply either an increased probability of pore opening or a modulator-induced stabilization of the open state channel dynamics.

In summary, the combination of bioinformatics tools plus pharmacological protocols successfully assisted a comprehensive understanding of the mechanism of P2X4R modulation by allosteric modulators. Three main conclusions highlight the perspectives posed by the present findings. (1) The molecular basis that account for the IVM P2X4R specificity compared to the related P2XRs family members. (2) The link of molecular pharmacology with structural biology, via the elucidation of the precise IVM allosteric site and the partial assignment of the Zn(II) site. (3) Channel pore dynamics and lateral fenestration enlargement by the allosteric modulators Zn(II) and IVM as a requirement for the increased ion flux, contrasting with the central pore expansion characteristic of Cys loop ligand-gated ion channels. Altogether, the combination of these methodologies allows visualizing the molecular events that support allosteric modulation, adding novel implications to the foundations of molecular pharmacology.

## Author contributions

VL: designed and performed in-silico experiments, analyzed data, and wrote the paper. FR and FG: performed in-vitro experiments and analyzed data. FM: analyzed in-silico data and wrote the paper. NB: analyzed data and wrote the paper; JH-T: supervised the investigation, analyzed data, and wrote the paper.

### Conflict of interest statement

The authors declare that the research was conducted in the absence of any commercial or financial relationships that could be construed as a potential conflict of interest.

## Supplementary material

Supplementary Figure 1 Grafical representation of hydrophobic environment related to the IVM putative TM domain allosteric binding site in P2XRs based on free energies of transfer from water to ethanol.

## References

[B1] Acuña-Castillo C. Coddou C. Bull P. Brito J. Huidobro-Toro J. P. (2007). Differential role of extracellular histidines in copper, zinc, magnesium and proton modulation of the P2X7 purinergic receptor. J. Neurochem. 101, 17–26. 10.1111/j.1471-4159.2006.04343.x 17394459

[B2] Acuña-Castillo C. Morales B. Huidobro-Toro J. P. (2000). Zinc and copper modulate differentially the P2X4 receptor. J. Neurochem. 74, 1529–1537. 10.1046/j.1471-4159.2000.0741529.x 10737610

[B3] Adelsberger H. Lepier A. Dudel J. (2000). Activation of rat recombinant alpha(1)beta(2)gamma(2S) GABA(A) receptor by the insecticide ivermectin. Eur. J. Pharmacol. 394, 163–170. 10.1016/S0014-2999(00)00164-3 10771281

[B4] Barrera N. P. Ormond S. J. Henderson R. M. Murrell-Lagnado R. D. Edwardson J. M. (2005). Atomic force microscopy imaging demonstrates that P2X2 receptors are trimers but that P2X6 receptor subunits do not oligomerize. J. Biol. Chem. 280, 10759–10765. 10.1074/jbc.M412265200 15657042

[B5] Brooks B. R. Brooks C. L. Mackerell A. D. Nilsson L. Petrella R. J. (2009). CHARMM: the biomolecular simulation program. J. Comput. Chem. 30, 1545–1614. 10.1002/jcc.21287 19444816 PMC2810661

[B6] Burnstock G. (2007). Purine and pyrimidine receptors. Cell. Mol. Life Sci. 64, 1471–1483. 10.1007/s00018-007-6497-0 17375261 PMC11149472

[B7] Coddou C. Acuña-Castillo C. Bull P. Huidobro-Toro J. P. (2007). Dissecting the facilitator and inhibitor allosteric metal sites of the P2X4 receptor channel: critical roles of CYS132 for zinc potentiation and ASP138 for cooper inhibition. J. Biol. Chem. 282, 36879–36886. 10.1074/jbc.M706925200 17962187

[B8] Coddou C. Lorca R. A. Acuña-Castillo C. Grauso M. Rassendren F. Huidobro-Toro J. P. (2005). Heavy metals modulate the activity of the purinergic P2X4 receptor. Toxicol. Appl. Pharmacol. 202, 121–131. 10.1016/j.taap.2004.06.015 15629187

[B9] Coddou C. Morales B. Gonzalez J. Grauso M. Gordillo F. Bull P. (2003a). Histidine 140 plays a key role in the inhibitory modulation of the P2X4 nucleotide receptor by copper but not zinc. J. Biol. Chem. 278, 36777–36785. 10.1074/jbc.M305177200 12819199

[B10] Coddou C. Morales B. Huidobro-Toro J. P. (2003b). Neuromodulator role of zinc and copper during prolonged ATP applications to P2X4 purinoceptors. Eur. J. Pharmacol. 472, 49–56. 10.1016/S0014-2999(03)01864-8 12860472

[B11] Coddou C. Yan Z. Obsil T. Huidobro-Toro J. P. Stojilkovic S. S. (2011). Activation and regulation of purinergic P2X receptor Channels. Pharmacol. Rev. 63, 641–683. 10.1124/pr.110.003129 21737531 PMC3141880

[B12] Cully D. F. Vassilatis D. K. Liu K. K. Paress P. S. Van der Ploeg L. H. Schaeffer J. M.. (1994). Cloning of an avermectin-sensitive glutamate-gated chloride channel from *Caenorhabditis elegans*. Nature 371, 707–711. 10.1038/371707a0 7935817

[B13] Gao C. Yu Q. Xu H. Zhang L. Liu J. Jie Y. (2015). Roles of the lateral fenestration residues of the P2X(4) receptor that contribute to the channel function and the deactivation effect of ivermectin. Purinergic Signal. 11, 229–238. 10.1007/s11302-015-9448-5 25847072 PMC4425721

[B14] Geary T. G. (2005). Ivermectin 20 years on: maturation of a wonder drug. Trends Parasitol. 21, 530–532. 10.1016/j.pt.2005.08.014 16126457

[B15] Hattori M. Gouaux E. (2012). Molecular mechanism of ATP binding and ion channel activation in P2X receptors. Nature 485, 207–212. 10.1038/nature11010 22535247 PMC3391165

[B16] He M. L. Zemkova H. Stojilkovic S. S. (2003). Dependence of purinergic P2X receptor activity on ectodomain structure. J. Biol. Chem. 278, 10182–10188. 10.1074/jbc.M209094200 12524445

[B17] Hibbs R. E. Gouaux E. (2011). Principles of activation and permeation in an anion-selective Cys-loop receptor. Nature 474, 54–60. 10.1038/nature10139 21572436 PMC3160419

[B18] Huidobro-Toro J. P. Lorca R. A. Coddou C. (2008). Trace metals in the brain: allosteric modulators of ligand-gated receptor channels, the case of ATP-gated P2X receptors. Eur. Biophys. J. 37, 301–314. 10.1007/s00249-007-0230-7 17972073

[B19] Jelinkova I. Vavra V. Jindrichova M. Obsil T. Zemkova H. W. Zemkova H. (2008). Identification of P2X4 receptor transmembrane residues contributing to channel gating and interaction with ivermectin. Pflugers Arch. 456, 939–950. 10.1007/s00424-008-0450-4 18427835

[B20] Jelínková I. Yan Z. Liang Z. Moonat S. Teisinger J. Stojilkovic S. S.. (2006). Identification of P2X4 receptor-specific residues contributing to the ivermectin effects on channel deactivation. Biochem. Biophys. Res. Commun. 349, 619–625. 10.1016/j.bbrc.2006.08.084 16949036

[B21] Jiang R. Taly A. Grutter T. (2013). Moving through the gate in ATP- activated P2X receptors. Trends Biochem. Sci. 38, 20–29. 10.1016/j.tibs.2012.10.006 23206935

[B22] Jorgensen W. L. Chandrasekha R. J. Madura J. D. Impey R. W. Klein M. I. (1983). Comparison of simple potential functions for simulating liquid water. J. Chem. Phys. 79, 926–935. 10.1063/1.445869

[B23] Karasawa A. Kawate T. (2016). Structural basis for subtype-specific inhibition of the P2X7 receptor. Elife 5:e22153. 10.7554/eLife.22153 27935479 PMC5176352

[B24] Kardos J. Kovács I. Hajós F. Kálman M. Simonyi M. (1989). Nerve endings from rat brain tissue release copper upon depolarization. A possible role in regulating neuronal excitability. Neurosci. Lett. 103, 139–144. 10.1016/0304-3940(89)90565-X 2549468

[B25] Kawate T. Michel J. C. Birdsong W. T. Gouaux E. (2009). Crystal structure of the ATP-gated P2X(4) ion channel in the closed state. Nature 460, 592–598. 10.1038/nature08198 19641588 PMC2720809

[B26] Kawate T. Robertson J. L. Li M. Silberberg S. D. Swartz K. J. (2011). Ion access pathway to the transmembrane pore in P2X receptor channels. J. Gen. Physiol. 137, 579–590. 10.1085/jgp.201010593 21624948 PMC3105519

[B27] Kay A. R. (2006). Imaging synaptic zinc: promises and perils. Trends Neurosci. 29, 200–206. 10.1016/j.tins.2006.02.004 16515810

[B28] Kay A. R. Tóth K. (2006). The influence of location of a fluorescent zinc-probe in brain slices on its response to synaptic activation. J. Neurophysiol. 95, 1949–1956. 10.1152/jn.00959.2005 16319204

[B29] Khakh B. S. Bao X. R. Labarca C. Lester H. A. (1999). Neuronal P2X transmitter- gated cation channels change their ion selectivity in seconds. Nat. Neurosci. 2, 322–330. 10.1038/7233 10204538

[B30] Kim S. Thiessen P. A. Bolton E. E. Chen J. Fu G. Gindulyte A.. (2016). PubChem substance and compound databases. Nucleic Acids Res. 44, D1202–D1213. 10.1093/nar/gkv951 26400175 PMC4702940

[B31] Köles L. Fürst S. Illes P. (2007). Purine ionotropic (P2X) receptors. Curr. Pharm. Des. 2368–2384. 10.2174/138161207781368747 17692007

[B32] Levitt M. (2001). The birth of computational structural. Nat. Struct. Biol. 8, 392–393. 10.1038/87545 11323711

[B33] Lorca R. A. Coddou C. Gazitúa M. C. Bull P. Arredondo C. Huidobro-Toro J. P. (2005). Extracellular histidine residues identify common structural determinants in the copper/zinc P2X2 receptor modulation. J. Neurochem. 95, 499–512. 10.1111/j.1471-4159.2005.03387.x 16190872

[B34] Lovell S. C. Davis I. W. Arendall W. B. III. de Bakker P. I. W. Word J. M. Prisant M. G.. (2002). Structure validation by Calpha geometry: phi, psi and Cβ deviation. Proteins 50, 437–450. 10.1002/prot.10286 12557186

[B35] Mackay L. Zemkova H. Stojilkovic S. S. Sherman A. Khadra A. (2017). Deciphering the regulation of P2X4 receptor channel gating by ivermectin using Markov models. PLoS Comput. Biol. 13:e1005643 10.1371/journal.pcbi.1005643 28708827 PMC5533465

[B36] Marquez-Klaka B. Rettinger J. Bhargava Y. Eisele T. Nicke A. (2007). Identification of an intersubunit cross-link between substituted cysteine residues located in the putative ATP binding site of the P2X1 receptor. J. Neurosci. 27, 1456–1466. 10.1523/JNEUROSCI.3105-06.2007 17287520 PMC6673578

[B37] Morris G. M. Goodsell D. S. Halliday R. S. Huey R. Hart W. E. (1998). Automated docking using a Lamarckian genetic algorithm and an empirical free energy function. J. Comput. Chem. 19, 1639–1662 10.1002/(SICI)1096-987X(19981115)19:14<1639::AID-JCC10>3.0.CO;2-B

[B38] Morris G. M. Huey R. Lindstrom W. Sanner M. F. Belew R. K. Goodsell D. S. (2009). Autodock4 and AutoDockTools4: automated docking with selective receptor flexibility. J. Comput. Chem. 16, 2785–2791. 10.1002/jcc.21256 PMC2760638 19399780

[B39] Nicke A. Bäumert H. G. Rettinger J. Eichele A. Lambrecht G. Mutschler E.. (1998). P2X1 and P2X3 receptors form stable trimers: a novel structural motif of ligand-gated ion channels. EMBO J. 17, 3016–3028. 10.1093/emboj/17.11.3016 9606184 PMC1170641

[B40] Nozaki Y. Tanford C. (1971). The solubility of amino acids and two glycine peptides in aqueous ethanol and dioxane solutions. establishment of a hydrophobicity scale. J. Biol. Chem. 246, 2211–2217. 5555568

[B41] Omura S. Crump A. (2004). The life and times of ivermectin - a success story. Nat. Rev. Microbiol. 2, 984–989. 10.1038/nrmicro1048 15550944

[B42] Peralta F. A. Huidobro-Toro J. P. (2016). Zinc as allosteric ion channel modulator: ionotropic receptors as metalloproteins. Int. J. Mol. Sci. 17:E1059. 10.3390/ijms17071059 27384555 PMC4964435

[B43] Phillips J. C. Braun R. Wang W. Gumbart J. Tajkhorshid E. Villa E.. (2005). Scalable molecular dynamics with NAMD. J. Comput. Chem. 26, 1781–1802. 10.1002/jcc.20289 16222654 PMC2486339

[B44] Popova M. Trudell J. Li K. Alkana R. Davies D. Asatryan L. (2013). Tryptophan 46 is a site for ethanol and ivermectin action in P2X4 receptors. Purinergic Signal. 9, 621–632. 10.1007/s11302-013-9373-4 23817978 PMC3889384

[B45] Priel A. Silberberg S. D. (2004). Mechanism of ivermectin facilitation of human P2X4 receptor channels. J. Gen. Physiol. 123, 281–293. 10.1085/jgp.200308986 14769846 PMC2217454

[B46] Roberts J. A. Allsoppa R. C. El Ajouz S. Vial C. Schmid R. Young M. T. (2012). Agonist binding evokes extensive conformational changes in the extracellular domain of the ATP- gated human P2X1 receptor ion channel. Proc. Natl. Acad. Sci. U.S.A. 109, 4663–4667. 10.1073/pnas.1201872109 22393010 PMC3311380

[B47] Sali A. Blundell T. L. (1993). Comparative protein modelling by satisfaction of spatial restraints. J. Mol. Biol. 234, 779–815. 10.1006/jmbi.1993.1626 8254673

[B48] Samways D. S. K. Khakh B. S. Dutertre S. Egan T. M. (2011). Preferential use of unobstructed lateral portals as the access route to the pore of human ATP-gated ion channels (P2X receptors). Proc. Natl. Acad. Sci. U.S.A. 108, 13800–13805. 10.1073/pnas.1017550108 21808018 PMC3158179

[B49] Samways D. S. Khakh B. S. Egan T. M. (2012). Allosteric modulation of Ca2+ flux in ligand-gated cation channel (P2X4) by actions on lateral portals. J. Biol. Chem. 287, 7594–7602. 10.1074/jbc.M111.322461 22219189 PMC3293559

[B50] Shen M. Y. Sali A. (2006). Statistical potential for assessment and prediction of protein structures. Protein Sci. 15, 2507–2524. 10.1110/ps.062416606 17075131 PMC2242414

[B51] Silberberg S. D. Li M. Swartz K. J. (2007). Ivermectin interaction with transmembrane helices reveals widespread rearrangements during opening of P2X receptor channels. Neuron 54, 263–274. 10.1016/j.neuron.2007.03.020 17442247

[B52] Smart O. S. Neduvelil J. G. Wang X. Wallace B. A. Sansom M. S. (1996). HOLE: a program for the analysis of the pore dimensions of ion channel structural models. J. Mol. Graph. 14, 354–360. 10.1016/S0263-7855(97)00009-X 9195488

[B53] Thauer R. K. Jungermann K. Decker K. (1977). Energy conservation in chemotrophic anaerobic bacteria. Bacteriol. Rev. 41, 100–180. 860983 10.1128/br.41.1.100-180.1977 PMC413997

[B54] Theodorson-Norheim E. (1987). Friedman and quade tests: BASIC computer program to perform nonparametric two-way analysis of variance and multiple comparisons of several related samples. Comput. Biol. Med. 17, 85–99. 10.1016/0010-4825(87)90003-5 3581810

[B56] Vassilatis D. K. Arena J. P. Plasterk R. H. Wilkinson H. A. Schaeffer J. M. Cully D. F.. (1997). Genetic and biochemical evidence for a novel avermectin-sensitive chloride channel in *Caenorhabditis elegans*. Isolation and characterization. J. Biol. Chem. 272, 33167–33174. 10.1074/jbc.272.52.33167 9407104

[B57] Viswanadhan V. N. Ghose A. K. Revankar G. R. Robins R. K. (1989). Atomic physicochemical parameters for three dimensional structure directed quantitative structure-activity relationships. 4. Additional parameters for hydrophobic and dispersive interactions and their application for an automated superposition of certain naturally occurring nucleoside antibiotics. J. Chem. Inf. Comput. Sci. 29, 163–172. 10.1021/ci00063a006

[B58] von Heijne G. Blomberg C. (1979). Trans-membrane translocation of proteins. The direct transfer model. Eur. J. Biochem. 97, 175–181. 10.1111/j.1432-1033.1979.tb13100.x 477664

[B59] Wiederstein M. Sippl M. J. (2007). ProSA-web: interactive web service for the recognition of errors in three-dimensional structures of proteins. Nucleic Acids Res. 35, W407–W410. 10.1093/nar/gkm290 17517781 PMC1933241

[B60] Zemkova H. Khadra A. Rokic M. B. Tvrdonova V. Sherman A. Stojilkovic S. S. (2015). Allosteric regulation of the P2X4 receptor channel pore dilation. Pflugers Arch. 467, 713–726. 10.1007/s00424-014-1546-7 24917516 PMC4526220

[B61] Zemkova H. Tvrdonova V. Bhattacharya A. Jindrichova M. (2014). Allosteric modulation of ligand gated ion channels by ivermectin. Physiol. Res. 63(Suppl. 1), S215–S224. 24564661 10.33549/physiolres.932711

[B62] Zemkova H. Yan Z. Liang Z. Jelinkova I. Tomic M. Stojilkovic S. S. (2007). Role of aromatic and charged ectodomain residues in the P2X(4) receptor functions. J Neurochem. 102, 1139–1150. 10.1111/j.1471-4159.2007.04616.x 17663752

