# Supplementary figures and images for: P2X4 Receptor in Silico and Electrophysiological Approaches Reveal Insights of Ivermectin and Zinc Allosteric Modulation
Source: Front Pharmacol. 2017 Dec 15;8:918. doi: 10.3389/fphar.2017.00918 (PMC5737101; doi:10.3389/fphar.2017.00918)

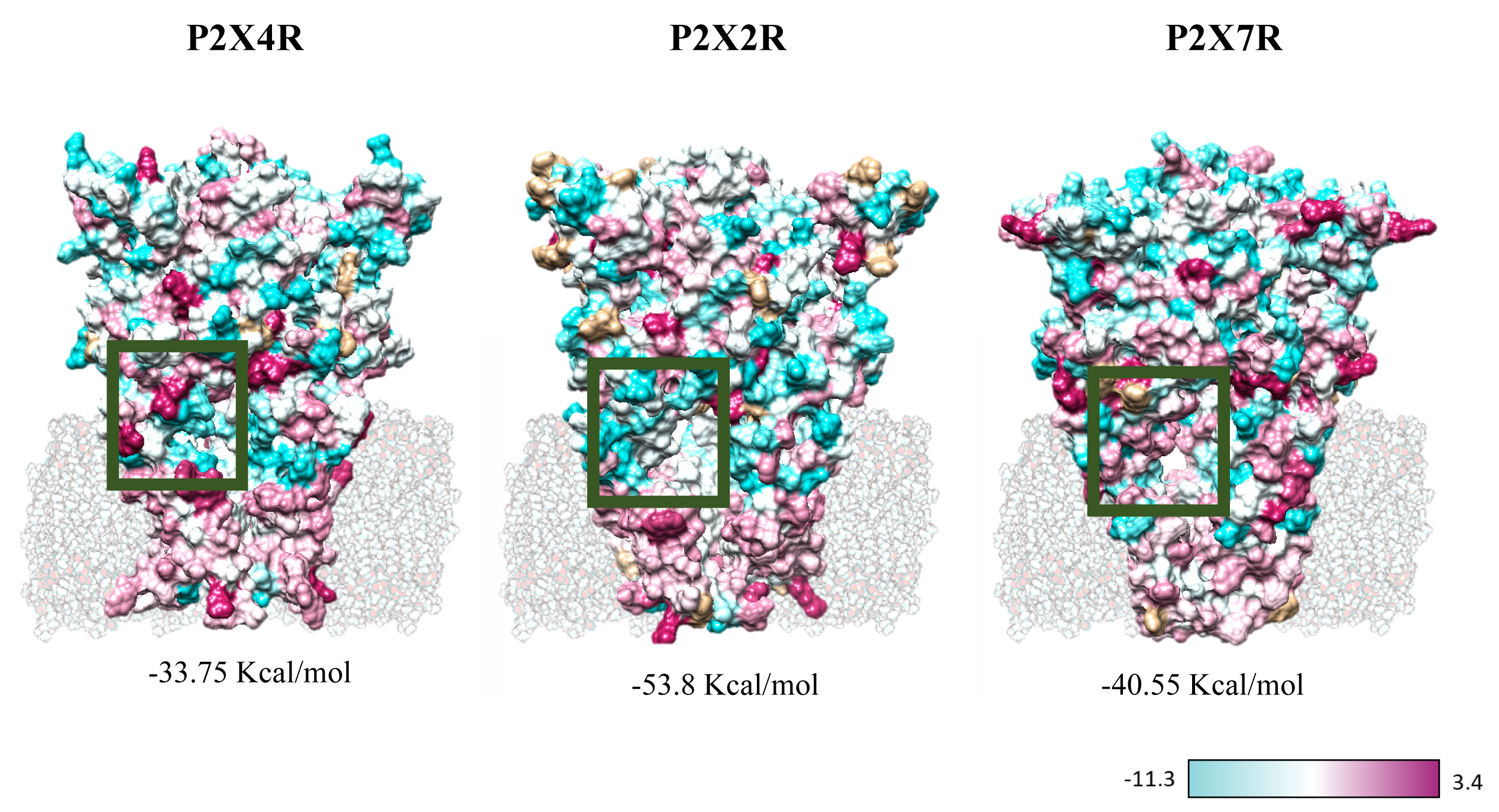

Supplement: Supplementary Figure 1 — Grafical representation of hydrophobic environment related to the IVM putative TM domain allosteric binding site in P2XRs based on free energies of transfer from water to ethanol. [file Image1.TIF]
